# Doping in Sports, a Never-Ending Story?

**DOI:** 10.15171/apb.2018.062

**Published:** 2018-11-29

**Authors:** Robert Alexandru Vlad, Gabriel Hancu, Gabriel Cosmin Popescu, Ioana Andreea Lungu

**Affiliations:** ^1^Department of Pharmaceutical Technology, Faculty of Pharmacy, University of Medicine and Pharmacy, Tîrgu MureŞ, Romania.; ^2^Department of Pharmaceutical Chemistry, Faculty of Pharmacy, University of Medicine and Pharmacy from Tîrgu MureŞ, Tîrgu MureŞ, Romania.; ^3^SC Sandoz SRL, Tîrgu MureŞ, Romania.; ^4^Cluj Municipal Clinical Hospital, Cluj-Napoca, Romania.

**Keywords:** Doping, Sports, Medication, Illicit substances

## Abstract

Through doping, we understand the use by athletes of substances prohibited by the antidoping agencies in order to gain a competitive advantage. Since sport plays an important role in physical and mental education and in promoting international understanding and cooperation, the widespread use of doping products and methods has consequences not only on health of the athletes, but also upon the image of sport. Thus, doping in sports is forbidden for both ethical and medical reasons.

Narcotics and analgesics, anabolic steroids, hormones, selective androgen receptor modulators are among the most frequently utilized substances.

Although antidoping controls are becoming more rigorous, doping and, very importantly, masking doping methods are also advancing, and these are usually one step ahead of doping detection techniques.

Depending on the sport practiced and the physical attributes it requires, the athletes will look for one or more of the following benefits of doping: recovering from an injury, increasing body recovery capacity after training, increasing muscle mass and strength, decreasing fat tissue, increasing endurance.

Finally, when we look once again at a doping scandal, amazed at how much animosity against those caught can exist; the question is: is it really such a disaster as presented by the media or a silent truth under our eyes, but which many of us have refused to accept?

## Introduction

Doping has become a key and complex issue in the sports world, which deserves serious consideration, as specialists are still striving to understand how and why it happens, and how to prevent it. "Sensational" revelations in the press reflect the gravity of a worrying situation resonating in most sports disciplines.^1^

Cases of doping compromise the credibility of performance in sport, the mediatized victories of some "arena heroes" becoming questionable and disputable. Nowadays some sporting disciplines seem to have managed to surpass the human limits and sometimes even the legal limits. The financial interests, the pressure to obtain better results, the media coverage of sports competitions and, last but not least, the human nature can explain this phenomenon.^2^

It is clear that in some disciplines such as athletics or cycling, human performance cannot improve endlessly. Nowadays, sports are no longer just sports; as sport become an industry, a business, a reason for political or national pride, and these facts can only lead to breaking any rules to win. Sometimes, consciously, camouflaged, with a network of specialists behind or on their own, some athletes think "maybe they won’t catch me"; because today sports mean sponsors, advertising contracts and money and for that some believe that any risk is worth taking. Even risks to their own health (often with huge and irreversible consequences) no longer matter.^3^

How does an athlete win a lot of money? From an important race and from sponsors. Where do organizers of sporting events have substantial prize money? From sponsors. Why do sponsors give substantial amounts of prizes? For publicity and to be associated with a first class sporting event where the best athletes participate. Who is watching a first class sports event? Everyone. The same people that will no longer watch the event when the athletes no longer offer the necessary show. The athletes today can be associated to the gladiators from Ancient Rome who were providing The commercial side of a sporting event is also an important matter. If people like the event in the modern arena, then the commercial success of the sporting event is assured and the sponsors are satisfied and will finance future events, thus providing funds for the organizers to give substantial prizes for the athletes.^4^

The doping phenomenon in sports is increasing and diversifying, as are the drugs used for doping. There is a permanent race among those who invent new doping methods and sports ethics organizations that are searching for more performant methods to detect them. Unfortunately, most of the times, those in the first category are always one step ahead.^5^

Improving scientific procedures used to detect prohibited substances is of course a necessity and also a challenge. Stricter legislation with the involvement of authorities is required to prevent the spread, marketing and use of such substances. Resolute action is required to restore fair-play throughout the sports industry and last but not least, the ethics and fair-play education of young athletes.^6^

The aim of this review is to gather and critically analyze recent developments and information regarding this sensitive issue, in order to offer a better understanding towards its foundation provided by previous research and to help develop practical strategies to effectively combat doping in sports.

## Doping from the beginning to the present day

Over time, there have been several definitions of doping. Beckmann's sports dictionary describes doping as the use of performance-increasing substances, which would place the athlete on a superior position than that he would normally have obtained.^7^

The first official definition of doping dates from 1963 and it was issued by the European Committee Council: "Doping represents the use of substances or physiological mediators, which are not normally present in the human body, introduced as an external aid to increase the athletes’ performance during a competition".^8^

According to the Anti-doping Convention of the European Council - "Doping in sports" means the administration or use of doping agents or doping methods by athletes. The doping agents or methods referred to are those doping agents which have been banned by the Anti-Doping Agency and which appear on a list of ineligible substances. "Athletes" are those persons normally participating in organized sports activities.^8^

Doping is not a modern term; in Norwegian mythology the use of performance/strength-increasing substances has been reported; as bufotenin, a substance known to increase the physical performance obtained from frogs skin or from Amanita mushrooms species.^9^

In ancient Greece, the use of prohibited substances was not discouraged, as specialists offered athletes various ingredients in order to increase physical performance; and this was considered absolutely normal, those who offered such substances being considered medical specialists in sports.^9^

Doping methods were used also in the Roman Empire, where racing horses were doped with various blends of substances aimed to increase their speed and stamina; also gladiators have been mentioned as users of strength-increasing agents.^9^

Doping was described in modern sports in the second half of the XIX century. During the Saint Louis marathon in 1904, Tom Hicks died as a result of using a mixture of cognac and strychnine. After multiple incidents in competitions, in 1928, the International Athletics Federation (IAF) became the first international federation to ban doping in athletic competitions; 32 years later anti-doping testing was implemented.^9^

Regarding the Olympics, the first official controls took place at the 1972 Olympic Games in Munich for conventional substances. Anabolic steroids were the first substances controlled at the 1976 Olympics in Montreal and as a consequence many athletes were disqualified and lost their medals. This lead to a decision by the International Olympic Committee (IOC), which stated that the results of doping tests should be made public within the competition.^10^

That was the beginning of an open fight that begins in the 1980s between those seeking and finding new doping substances that are not yet on the antidoping list and the authorities that try to detect these substances. It is clear, however, that between these two sides there is a gap in favor of those interested in cheating. Introducing anti-doping controls outside competitions was a new milestone in the anti-doping campaign in 1989.^11^

In modern professional sports, many athletes have been tested positive with forbidden substances, perhaps the most publicized case being that of canadian Ben Johnson, the famous 100 meters runner for the use of anabolic steroids. It was the first doping scandal in the history of the Olympic Games, which led to Johnson’s suspension for two years and then for life, because he tested positive again in 1993.^12^

After the fall of the Iron Curtain, information about industrial, systematic and scientific doping from the former German Democratic Republic and in general from the comunist states started appearing, with dozens of athletes experimenting the side-effects after the end of their career. This information revealed a negative aspect of sports history, unscrupulously used as a propaganda tool to demonstrate the superiority of the socialist society in which the athlete and his health represented nothing.^13^

Currently, doping is considered as any violation of the following rules: the use or attempt to use a forbidden substance or a prohibited method, refusal for sampling after receiving an invitation to doping control in accordance with anti-doping rules, avoidance of sampling, falsification or attempt to falsify any part of the doping control, possession of prohibited substances and / or methods, trafficking or attempted trafficking of any prohibited substance and / or methods.^14^

## Doping today

Depending on the country's legislation, doping substances can be bought from pharmacies / supplement stores or, most commonly, from the black market. For a substance or performance improvement method to be classified as doping, it must meet at least two of the following three criteria: to improve performance, to present a hazard to the health of the athlete and to violate the spirit of sport. Other methods of improving performance such as blood transfusions are also included in the doping category.

The number of doping substances is very high, and their individual cataloging is not the purpose of this article. Instead, we can make a general classification according to how they act. A classification from S0 to S9 (Table 1) for prohibited substances and from M1 to M3 (Table 2) for prohibited methods has been developed.

**Table 1:** Banned substances both during and outside the competition^15^

**S0. Substances that have not been placed on the market**	Retired drugs such as sibutramine;	Designer substances: tetrahydrogestrinone	Drugs used in veterinary medicine
**S1.Anabolic agents**	Exogenous anabolic steroids: androstendiol and gestrinone	Endogenous anabolic steroids with exogenous administration: dihydrotestosterone, testosterone	Other anabolic agents: tibolone, zilpaterol, zeranol
**S2. Peptide hormones and growth factors**	Erythropoiesis stimulating agents: erythropoietin, darboietin	Luteinizing hormone in men; choriogonadotrophin	Corticotrophins, Growth Hormones. Insulin-like growth factor 1 (IGF 1)
**S3. Beta 2 agonists**	Salbutamol-1600 µg /24h	Formoterol 54 µg/ 24h	Clenbuterol
**S4. Hormones and metabolic modulators**	Aromatase inhibitors: aminoglutethimide	Metabolic mediators: insulin	-
**S5. Diuretics and other masking agents**	Masking agents: glycerols, plasma substitutes	Diuretics: Acetazolamide, Furosemide, Indapamide	-
**S6. CNS stimulants**	Nonspecific stimulants: amfepramone, fenfluramine	Specific stimulants: adrenaline, ephedrine, pseudoephedrine	-
**S7. Narcotics**	Buprenorphine, fentanyl	Metadone, morphine	-
**S8. Cannabis extracts**	Cannabis, hashish	Tetrahydrocannabinol	-
**S9. Corticosteroids**	Cortizon, Hydrocortisone	Prednison, Metilprednisolone	-

**Table 2:** Prohibited methods^15^

**M1. Manipulation of blood and its components**	Administration of products containing red blood cells in the circulatory system	Increasing the amount of oxygen or its transport
**M2.Physical and chemical handling**	Altering the integrity and validity of the sample collected during anti-doping control	Intravenous infusions or injections of more than 50 mL for 6 hours
**M3. Genetically doping**	Transfer of polymers of nucleic acids or their analogs	Use of normal or genetically modified cells

Since 2004, the World Anti-Doping Agency (WADA) has annually updated their Code and related documents that outline the ofﬁcial international anti-doping standards.

## Substances which are not on the list of prohibited substances with possible doping effect

One of the substances that are currently extensively studied for doping potential is paracetamol, a substance commonly used as an analgesic and antipyretic. It has been noticed that in the case of cyclists, the athletes performances have been improved. So if in the case of cyclists it can increase performance, by lowering body temperature; why couldn’t it be used for athletes practicing marathon, or athletes who run the 5000 and 10000 meters distances ?^16^

Some herbal extracts were suspected to have doping effects, so the ginseng root was tested to detect possible performance enhancing effects, but according to studies conducted on athletes under the supervision of the IOC, no positive tests were observed. However, it is specified that due to contamination with other doping substances, the tests could be positive, due to which the nutraceuticals should be carefully checked prior to use, in order to prevent possible disqualification from competitions.^17^

Studies have also been conducted to see whether NSAIDs, diclofenac and ibuprofen, both being non-selective COX non-steroidal anti-inflammatory drugs, could have an effect on the testosterone / glucuronidated epitestosterone ratio, but the results did not reveal any modification.^18^

## Substances that are not forbidden but can increase the performance of the athlete

L-carnitine is an endogenous compound, an aminoacid synthesized in the liver and kidneys from lysine and methionine, two essential amino acids. It can be found especially in food of animal origin, but also in plants such as soy beans, although in much smaller quantities. L-carnitine administration increases the HDL cholesterol fraction, and has neuroprotective properties in Alzheimer's disease. For athletes, the use of L-carnitine is based on the release of energy from lipids, saving a part of the glycogen from the muscles.^19^

Arginine is a semi-essential amino acid that could be used to increase performance, because of NO (nitrogen monoxide) release and the formation of citrulline, NO having a vasodilatory effect. Athletes can use arginine to increase physical performance, muscle mass and also their resistance in high effort.^20^

Hydroxycitric acid is a substance often found in food supplements and it can be extracted from species such as Hibiscus sabdariffa or Garcinia cambodgia. It was reported to be used for weight loss, but according to clinical trials, it does not have lipolysis effects.^19^

Tyrosine is an essential amino acid that cannot be synthesized by the body and should be obtained through careful nutrition. It can also be used by athletes, with many beneficial effects such as reducing fat, controlling appetite. However, it is a dopamine precursor and so people with mental disorders or hyperthyroidism should not use it, as well as people with high risk of skin cancer because this amino acid leads to increased melatonin secretion. Another aspect to be considered is the period of the day when it is administered, because it is a precursor of adrenaline and noradrenaline that can cause stimulation of the nervous system.^21^

Other amino acids or derivatives used to increase muscle strength and endurance are: carnozine, citrulline, glutamine, glycine and taurine. Taurine and carnosine have particular effects, being used as energizing substances.

## Substances that are dopant only if certain doses are exceeded

There are some pharmacological classes of substances that have a quantitative upper limit, so can be used only in very small amounts, as: central nervous system stimulants such as caffeine and beta 2 selectives such as salbutamol or fenoterol.

Caffeine can be considered as a dopant substance due to its effects: slight bronchodilatation, which is beneficial for athletes participating in endurance races, and also increases the diuresis which can be beneficial if an athlete is doped and wants to rapidly eliminate the other drug in their body. Other effects of caffeine are: cerebral vasoconstrictor, increases gastric acidity and also the appetite. An athlete is considered doped when the urine concentration of caffeine is above 12 μg/mL.^22^

Most beta 2 selective substances are banned from competitions, but there are exceptions such as salbutamol, which has a maximum inhalation dose of 1.6 mg/24h. If salbutamol is present in a concentration higher than 1000 ng/mL in urine the athlete can be considered as doped. Formoterol is a substance used in asthma and it is in the same category as salbutamol. The dose of inhaled formoterol is 54 μg/ 4h, and urine concentration should not exceed 40 ng/mL, otherwise the athlete is sanctioned according to the rules.^18^

Specific central nervous system stimulants are substances that also have thresholds, ephedrine and methylefedrine are prohibited when the concentration reaches values higher than 10 μg/mL, pseudoephedrine is prohibited when concentrations are greater than 150 μg/mL. Adrenaline is not forbidden when used locally in nasal or ophthalmic administration.^23^

Other substances that have a superior limit, that can lead to the elimination of the athlete from the competition are: bupropion, nicotine, pipradol, phenylephrine and phenylpropanolamine.

## Substances subject to a monitoring program

There are three classes of substances part of a monitoring program: central nervous system stimulants such as bupropion, nicotine, phenylephrine, phenylpropanolamine, sinephrine and pipradrol; narcotics: hydrocodone, tramadol, talpentadol; and glucocorticoids, banned in competition through all ways of administration. Also, telmisartan, a angiotensin II antagonist class on AT1 receptors and meldonium substance used in angina pectoris, can be included in the same category. Central nervous system stimulants as well as narcotics will not be used in competitions, while glucocorticoids, meldonium and telmisartan are banned both outside and in competitions.^23^

Due to the fact that methylmorphine (codeine) converts approximately 10% into morphine, the codeine/morphine ratio should also be checked and is only monitored in competitions.^19^

## Conclusion

The fight against doping continues, but anti-doping agencies will always be one step behind manufacturers of new undetectable substances with pharmacological properties similar to those already available on the market.

Much of the substances used today can be easily detected, but the development of new, cheaper and faster methods could help the Anti-Doping Federation.

The existence of rules and Codes, as well as Anti-Doping Procedures and Biological Passports make doping more and more difficult to achieve.

Another major factor that can lead to doping is the financial side, good results get sponsors and publicity, for some athletes being more than enough motivation for doping.

Injuries are another reason why many athletes endanger their "clean" athletes' status, their will to return to competition can lead to compromises that can end their career.

Coaches have an important role in athlets’ doping, most of the time, they are responsible for the illegal actions of athletes by offering them the forbidden substances or by acquainting them with people who are involved in doping. There are also athletes who do not know the utility of a substance or if it is on the forbidden list and with their doctor’s recommendation they use the substance which may be on the forbidden list. Another interesting case is that of food supplements purchased from unauthorized sites on the Internet. By having good ads with a convincing message, these supplements can be bought by an athlete. Unfortunately there is no organization to determine the composition of these food supplements, so when an athlete decides to use them, he is taking the risk of potential doping.

It is important to note that from the legal point of view, the athlete is 100% responsible for the substances that enter his body. Thus, if the athlete ingests accidentally an forbidden substance, he is still responsible for it.

Carrying out anti-doping controls both in and out of competitions is a benefit for athletes who do not use banned substances, the number of athletes who have been positively detected outside competitions is much higher than those who are found doped in competitions.

Physicians should pay attention when prescribing different substances, as well as pharmacists who release the medication. By releasing a drug on the list of prohibited substances, the athlete may be disqualified, so the regulations and the list of prohibited substances should be carefully studied before prescribing a medicinal product.

The effects it has on the body is also an important topic when discussing about doping. For example, artificial testosterone leads to stopping endogenous production of natural testosterone in the body. The difference is that today's doping substances are safer than they were 40-50 years ago, when some athletes died because of them. In fact, many steroids are of medical use today and are administered to patients who have undergone difficult operations and need faster recovery.

Athletes who use different medication and have the consent of physicians should be careful to declare the use of such substances so that if the athlete is positively detected with it, the authorities know that the substance is needed to improve their health condition.

Current legislation is not very severe, perhaps if the repercursions of being positive with illegal substances were higher, violation of rules would not be so common. Athletes should be educated about doping, and about the side and adverse effects of the use of the various prohibited substances, with the aim of educating athletes to prevent the doping phenomenon.

To minimize the phenomenon of doping, information and prevention programs, starting with athletes at a young age, and involving other stakeholders (e.g. the athletes’ doctors, coaches or family), are necessary to establish and maintain correct attitudes and behaviors.

Finally, we can conclude that taking into account the human nature and the social and economic implications of professional sports, the end of doping in sports is most likely an unrealistic term.

## Ethical Issues

Not applicable.

## Conflict of Interest

The authors declare that there are no conflicts of interest.
